# Patients with an Open Abdomen in Asian, American and European Continents: A Comparative Analysis from the International Register of Open Abdomen (IROA)

**DOI:** 10.1007/s00268-022-06733-4

**Published:** 2022-11-03

**Authors:** Maria Grazia Sibilla, Camilla Cremonini, Mattia Portinari, Paolo Carcoforo, Dario Tartaglia, Enrico Cicuttin, Serena Musetti, Silvia Strambi, Massimo Sartelli, Margherita Koleva Radica, Fausto Catena, Massimo Chiarugi, Federico Coccolini, IROA Study Group, Giulia Montori, Fracensco Salvetti, Ionut Negoi, Monica Zese, Savino Occhionorelli, Sergei Shlyapnikov, Michael Sugrue, Zaza Demetrashvili, Daniele Dondossola, Orestis Ioannidis, Giuseppe Novelli, Cristina Frattini, Mirco Nacoti, Desmond Khor, Kenji Inaba, Demetrios Demetriades, Torsten Kaussen, Asri Che Jusoh, Wagih Ghannam, Boris Sakakushev, Ohad Guetta, Agron Dogjani, Stefano Costa, Sandeep Singh, Dimitrios Damaskos, Arda Isik, Kuo-Ching Yuan, Francesco Trotta, Stefano Rausei, Aleix Martinez-Perez, Giovanni Bellanova, Vinicius Cordeiro Fonseca, Fernando Hernández, Athanasios Marinis, Wellington Fernandes, Martha Quiodettis, Miklosh Bala, Andras Vereczkei, Rafael Curado, Gustavo Pereira Fraga, Bruno M. Pereira, Mahir Gachabayov, Guillermo Perez Chagerben, Miguel Leon Arellano, Sefa Ozyazici, Gianluca Costa, Tugan Tezcaner, Matteo Porta, Yousheng Li, Faruk Karateke, Dimitrios Manatakis, Federico Mariani, Federico Lora, Ivan Sahderov, Boyko Atanasov, Sergio Zegarra, Luca Fattori, Rao Ivatury, Jimmy Xiao, Offir Ben-Ishay, Andrey Zharikov, Vincent Dubuisson

**Affiliations:** 1 Department of Surgery, Unit of General Surgery, University Hospital of Ferrara and University of Ferrara, Ferrara, Italy; 2 General Emergency and Trauma Surgery, Pisa University Hospital, Via Paradisia,1, 56124 Pisa, Italy; 3 General Surgery, Macerata Hospital, Macerata, Italy; 4 Emergency Surgery, Parma University Hospital, Parma, Italy

## Abstract

**Background:**

International register of open abdomen (IROA) enrolls patients from several centers in American, European, and Asiatic continent. The aim of our study is to compare the characteristics, management and clinical outcome of adult patients treated with OA in the three continents.

**Material and methods:**

A prospective analysis of adult patients enrolled in the international register of open abdomen (IROA). Trial registration: NCT02382770.

**Results:**

1183 patients were enrolled from American, European and Asiatic Continent. Median age was 63 years (IQR 49–74) and was higher in the European continent (65 years, *p* < 0.001); 57% were male. The main indication for OA was peritonitis (50.6%) followed by trauma (15.4%) and vascular emergency (13.5%) with differences among the continents (*p* < 0.001). Commercial NPWT was preferred in America and Europe (77.4% and 52.3% of cases) while Barker vacuum pack (48.2%) was the preferred temporary abdominal closure technique in Asia (*p* < 0.001). Definitive abdominal closure was achieved in 82.3% of cases in America (fascial closure in 90.2% of cases) and in 56.4% of cases in Asia (*p* < 0.001). Prosthesis were mostly used in Europe (17.3%, *p* < 0.001). The overall entero-atmospheric fistula rate 2.5%. Median open abdomen duration was 4 days (IQR 2–7). The overall intensive care unit and hospital length-of-stay were, respectively, 8 and 11 days (no differences between continents). The overall morbidity and mortality rates for America, Europe, and Asia were, respectively, 75.8%, 75.3%, 91.8% (*p* = 0.001) and 31.9%, 51.6%, 56.9% (*p* < 0.001).

**Conclusion:**

There is no uniformity in OA management in the different continents. Heterogeneous adherence to international guidelines application is evident. Different temporary abdominal closure techniques in relation to indications led to different outcomes across the continents. Adherence to guidelines, combined with more consistent data, will ultimately allow to improving knowledge and outcome.

## Introduction

The Open abdomen (OA) technique allows managing complex surgical situations in a damage control strategy, and to prevent or treat abdominal compartment syndrome (ACS) [1]. Main indications to OA are intra-abdominal infections, trauma, pancreatitis, and vascular emergencies. Several temporary abdominal closure techniques (TACTs) exist. OA has many advantages but as a counterpart, patients are at risk of developing complications, such as entero-atmospheric fistula (EAF), severe intestinal adhesions syndrome (frozen abdomen), formation of abscesses and consequences of a reduced rates of definitive fascial closure.

In the last 10 years, OA use has increased worldwide [2], therefore in 2015, the International register of open abdomen (IROA) was initiated [3] to overcome the lack of evidence-based data related to its indications, management and outcomes. Several centers from American, European and Asiatic continent contributed to the register. A number of publications, derived from IROA, investigated the different aspects of the OA management [4–7]. Therefore, in 2018 the WSES provided the international guidelines with the purpose to standardize the management of the OA as much as possible [8].

The aim of this study is to compare the characteristics, management and clinical outcome of adult patients treated with OA in American, European and Asiatic Continent.

## Material and methods

Data came from the IROA, the prospective observational international cohort study that enrolled patients treated with open abdomen worldwide. The registry is recorded on a web platform (Clinical Registers®) through a dedicated website (www.clinicalregisters.org) according to the study protocol, approved by the coordinating center Ethical Committee and registered to ClinicalTrials.gov (ClinicalTrials.gov Identifier: NCT02382770). A detailed description of the study protocol is available at www.clinicalregisters.org/IROA.

In this study, we included only adult patients (older than 16 years old) with an OA treatment and data were collected from May 2015 to September 2020.

Patients were divided into three subgroups according to the geographical area of their enrolling centers: the American, the European and the Asiatic continent.

Data collected for each patient included: demographical data, comorbidities, indication for treatment, type of temporary abdominal closure technique (TACT) and duration of the treatment, rates of primary fascial closure, type of definitive closure, rates of fistula and other complications, length of hospitalization, mortality before and after closure. Open abdomen indications were divided into seven groups: peritonitis, trauma, pancreatitis, ischemia, vascular emergencies and hemorrhage, post-operative ACS, trauma and other. “Other” included burns, caustic ingestion, massive resuscitation, occlusion and transplant. TACTs were divided in six subgroups (negative pressure wound therapy (NPWT), NPWT with dynamic tension, Wittmann patch, skin closure, Bogotà bag and Barker vacuum pack). Moreover, groups were divided into NPWT techniques (Barker vacuum pack, NPWT and NPWT with fascial traction) and non-NPWT (Bogotà bag, skin closure and Wittmann patch). Definitive closure was defined as fascia or skin closure.

### Statistical analysis

Data extracted from the IROA were analyzed using SPSS Statistics 23 (SPSS Inc., Chicago, IL). Descriptive statistics were calculated for all clinical variables described; for all the used tests, statistical significance level was set at the conventional *p* < 0.05. Continuous variables are represented as median and interquartile range (IQR); categorical data were expressed as proportions and percentages. The Kruskall-Wallis test was used to compare continuous variables. Pearson’s chi-squared test or Fisher exact test were used to compare categorical variables.

## Results

**Fig. 1:**
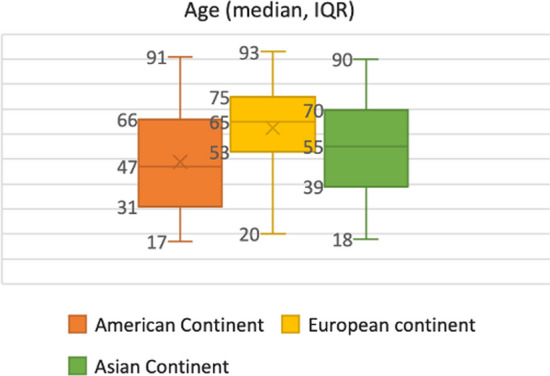
Median age (years, IQR) across the continents (*p* < 0.001)

A total number of 1183 patients were prospectively enrolled from America, European and Asiatic continents. The characteristics of the study population are summarized in Table 1. The median age of enrolled patients was 63 years (IQR 49–79) and 57% were male. The majority of patients enrolled in the American continent had an age between 16 and 40 years (40.2%); in Europe, the age’s range was between 60 and 80 (48.9%), whereas in Asia the distribution was more homogeneous. Patients enrolled from Europe were older compared to the other two continents (median age: 65 [IQR 53–75]; *p* < 0.001, Fig. 1). The median body mass index (BMI) was 26.1 (IQR 23.2–29.4) and median BMI distribution was significant different among continents (higher in the American continent, *p* = 0.049); however, due to the different BMI cutoff used to define obesity in the Asiatic continent (BMI > 25), obesity was a more common comorbid condition among Asiatic patients with 61.8% of cases involved (*p* < 0.001). Worldwide, the ASA (American Society of Anesthesiologists) score ≤ III was the most represented but, stratified by continents, the ASA ≤ III score prevailed in America instead ASA ≥ 4 score was more frequent in Europe.

**Table 1:** Demographics

	Total N = 1183	American continent N = 124 (10.5)	European continent N = 949 (80.2)	Asiatic continent N = 110 (9.3)	*P* value
Age (years), median (IQR)	63 (49 – 74)	47 (31 – 66)	65 (53 – 75)	55 (39 – 70)	**< 0.001**
Age > 65 y, n (%)	561 (47.4)	32 (26.2)	488 (51.8)	41 (37.6)	**< 0.001**
Age Class*, n (%)					**< 0.001**
16–40 y	179 (15.1)	49 (40.2)	101 (10.7)	29 (26.6)	
41–60 y	342 (28.9)	33 (27.0)	276 (29.3)	33 (30.3)	
61–80 y	534 (45.2)	34 (27.9)	461 (48.9)	39 (35.8)	
> 80 y Missing 10 (0.8)	118 (10.0)	6 (4.9)	104 (11.0)	8 (7.3)	
*Gender (n, %)*					**< 0.001**
Women	509 (43.0)	39 (31.5)	437 (46.0)	33 (30.0)	
Men	674 (57.0)	85 (68.5)	512 (54.0)	33 (70.0)	
BMI (kg/m2), median (IQR)	26.1 (23.2 – 29.4)	27.8 (23.4 – 31.5)	25.6 (23.2 – 29.4)	26.5 (23.2 – 29)	**0.049**
*ASA*					**< 0.001**
ASA I	67 (5.7)	9 (7.3)	44 (4.6)	14 (12.7)	
ASA II	191 (16.1)	29 (23.4)	134 (14.1)	28 (25.5)	
ASA III	386 (32.6)	47 (37.9)	315 (33.2)	24 (21.8)	
ASA IV	417 (35.2)	38 (30.6)	339 (35.7)	40 (36.4)	
ASA V	122 (10.3)	1 (0.8)	117 (12.4)	4 (3.6)	
Comorbidities**(n, %)					
AAA	61 (5.2)	4 (3.3)	49 (5.6)	8 (7.3)	0.423
Cancer	282 (23.8)	12 (9.9)	251 (28.8)	19 (17.3)	**< 0.001**
Cardiomyopathy	373 (31.5)	30 (24.8)	317 (36.3)	26 (33.8)	**0.003**
Diabetes mellitus	181 (15.3)	22 (18.2)	135 (15.5)	24 (21.8)	0.205
Hepatopathy	89 (7.5)	12 (9.9)	69 (7.9)	8 (7.3)	0.689
Immunological disorder	39 (3.3)	3 (2.5)	32 (3.7)	4 (3.6)	0.803
Immunosuppression	63 (5.3)	9 (7.4)	49 (5.6)	5 (4.5)	0.639
Nephropathy	131 (11.1)	17 (14.0)	102 (11.7)	12 (10.9)	0.711
Neurological disorder	91 (7.7)	7 (5.8)	77 (8.8)	7 (6.4)	0.417
None	175 (14.8)	37 (30.6)	117 (13.4)	21 (19.1)	**< 0.001**
Obesity	290 (24.5)	38 (30.6)	184 (20.9)	68 (61.8)	**< 0.001**
Other	230 (19.4)	30 (24.8)	189 (21.6)	11 (10.0)	**0.009**
Pneumological disorder	152 (12.8)	10 (8.3)	135 (15.5)	7 (6.4)	**0.006**
Presence of ileostomy	31 (2.6)	3 (2.5)	26 (3.0)	2 (1.8)	0.770
Presence of colostomy	30 (2.5)	1 (0.8)	25 (2.9)	4 (3.6)	0.353
Presence of urostomy	12 (1.0)	1 (0.8)	11 (1.3)	0	0.861
Remote infection	65 (5.5)	13 (10.7)	39 (4.5)	13 (11.8)	**0.001**
Smoking Missing 79 (6.7)	169 (14.3)	12 (9.9)	130 (14.9)	27 (24.5)	**0.006**

Bold characters indicate statistically significant values

BMI—body mass index, ASA—(American Society of Anesthesiologists) score

*Missing information of 10 patients; **Missing information of 79 patients

**Fig. 2:**
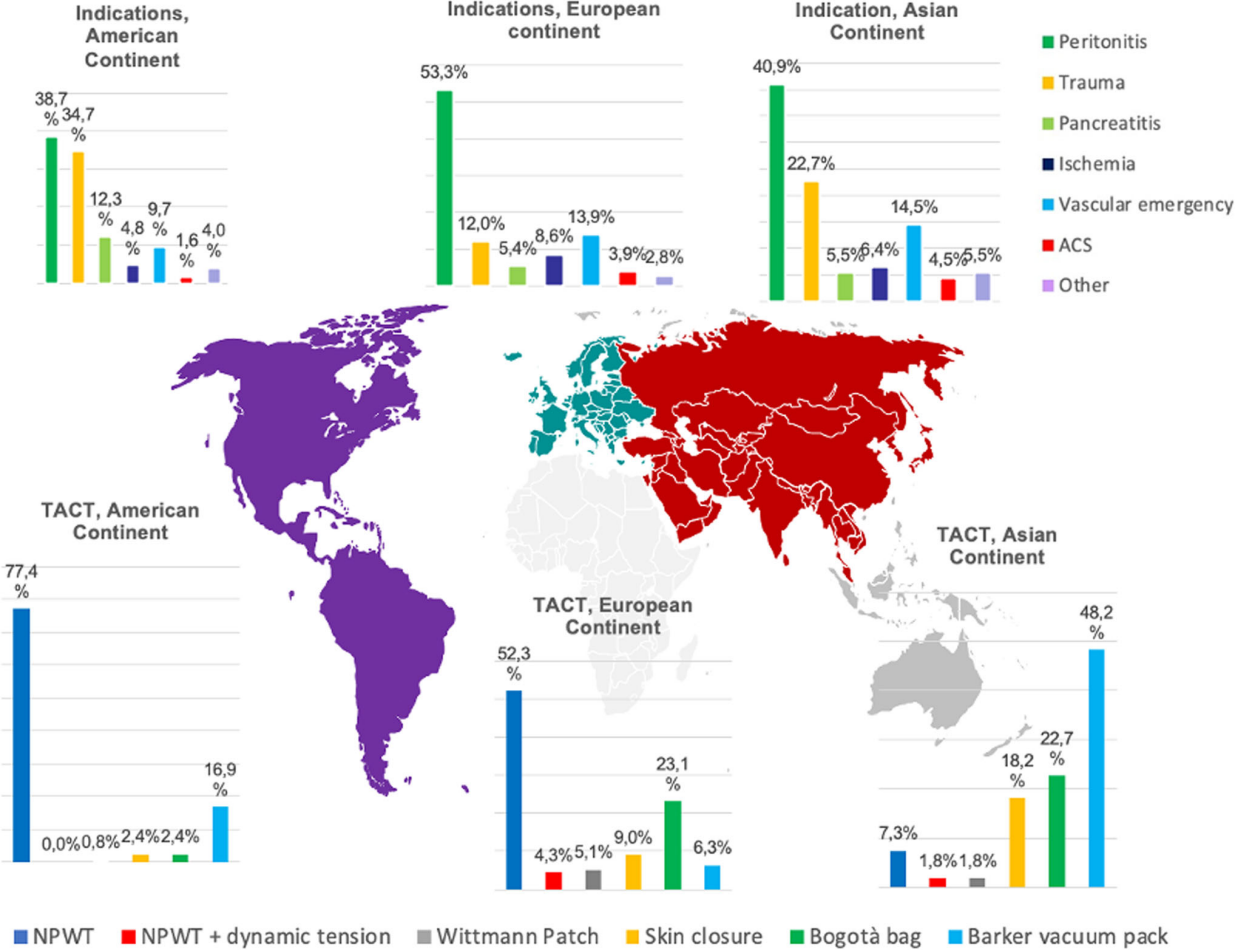
Distribution across American, European and Asian continent of Indications and Temporary Abdominal Closure Technique (TACT); *ACS* abdominal compartment syndrome; *NPWT* negative pressure wound therapy

**Fig. 3:**
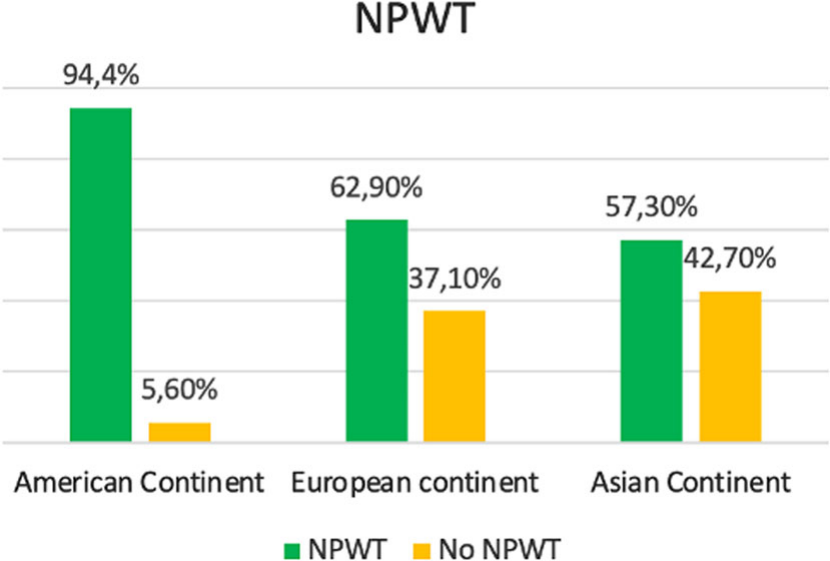
Use of NPWT across the continents

As shown in Table 2, the overall principal indication for OA was peritonitis followed by trauma and vascular emergencies but there were statistically significant differences among continents (*p* < 0.001). In America, peritonitis (38.7%) and trauma (34.7%) had a similar distribution, while in the European and the Asiatic continent prevailed peritonitis. Abdominal compartment syndrome (ACS) was an OA indication in 3.7% of patients. As regards the monitoring of intra-abdominal pressure (IAP), the 62.1% of the patients did not show intra-abdominal hypertension (IAH) before surgery, but only in Asiatic continent 29.8% of patients presented and IAH of GRADE I. The highest value of Injury Severity Score (ISS) of trauma treated with OA was in Asiatic continent (30, IQR 18–47.5) while the lowest was in America (21, IQR 10–36) (*p* = 0.003). Figure 2 shows the distribution of indication for OA and TACT across the three continents. The most adopted TACT was the commercial NPWT (54.4%) followed by the Bogota bag (20.9%) and Barker vacuum pack (11.3%). As shown in Table 3, surgeons in America and Europe preferred NPWT (77.4% and 52.3%, respectively) while in Asiatic continent used Barker in 48.2% of cases and a commercial NPWT in the 9.1% of cases (*p* < 0.001). Figure 3 summarizes the use of NPWT across the continents. In the American continent, definitive closure of the abdomen was obtained in 82.3% of cases with closure of the fascia obtained in 90.2% of cases while in the Asiatic continent it was obtained in 56.4% of cases. In the European continent, there was a major use of prosthesis (17,3%) to close the fascia, with biological mesh utilized in 10.6% of patients. The entero-atmospheric fistula rate (EAF) was globally 2.5% without significant differences among continents; data regarding this specific aspect were missing for 295 (24.9%) patients.

**Table 2:** Clinical data

	Total N = 1183	American continent N = 124 (10.5)	European continent N = 949 (80.2)	Asiatic continent N = 110 (9.3)	*P* value
*OA Indication (n, %)*					**< 0.001**
Peritonitis	599 (50.6)	48 (38.7)	506 (53.3)	45 (40.9)	
Trauma	182 (15.4)	43 (34.7)	114 (12.0)	25 (22.7)	
Pancreatitis	65 (5.5)	8 (12.3)	51 (5.4)	6 (5.5)	
Ischemia	95 (8.0)	6 (4.8)	82 (8.6)	7 (6.4)	
Vascular emergency	160 (13.5)	12 (9.7)	132 (13.9)	16 (14.5)	
ACS	44 (3.7)	2 (1.6)	37 (3.9)	5 (4.5)	
Other	38 (3.2)	5 (4.0)	27 (2.8)	6 (5.5)	
IAH grade *(n, %)					**< 0.001**
No IAH	735 (62.1)	46 (65.7)	639 (73.0)	50 (48.1)	
Grade I (12–15 mmHg)	125 (10.6)	6 (8.6)	88 (10.1)	31 (29.8)	
Grade II (16–20 mmHg)	81 (6.8)	8 (11.4)	64 (7.3)	9 (8.7)	
Grade III (21–25 mmHg)	76 (6.4)	2 (2.9)	67 (7.7)	7 (6.7)	
Grade IV (> 25 mmHg)	32 (2.7)	8 (11.4)	17 (1.9)	7 (6.7)	
ISS, median (IQR)	29 (20–41.3)	21 (10–36)	29.5 (24–42.3)	30 (18–47.5)	**0.003**

Bold characters indicate statistically significant values

ACS—Abdominal compartment syndrome; IAH—intra-abdominal hypertension; ISS—Injury Severity Score

*Missing information of 139 patients

**Table 3:** TACTs and OA closure

	Total N = 1183	American Continent N = 124 (10.5)	European Continent N = 949 (80.2)	Asiatic Continent N = 110 (9.3)	*P* value
*TACT*					**< 0.001**
NPWT	600 (50.7)	96 (77.4)	496 (52.3)	8 (7.3)	
NPWT + dynamic tension	43 (3.6)	0	41 (4.3)	2 (1.8)	
Wittmann Patch	51 (4.3)	1 (0.8)	48 (5.1)	2 (1.8)	
Skin closure	108 (9.1)	3 (2.4)	85 (9.0)	20 (18.2)	
Bogotà bag	247 (20.9)	3 (2.4)	219 (23.1)	25 (22.7)	
Barker vacuum pack	134 (11.3)	21 (16.9)	60 (6.3)	53 (48.2)	
*NPWT*					**< 0.001**
Yes	777 (65.7)	117 (94.4)	597 (62.9)	63 (57.3)	
No	406 (34.3)	7 (5.6)	352 (37.1)	47 (42.7)	
*Commercial NPWT*					**< 0.001**
yes	643 (54.4)	96 (82.1)	537 (89.9)	10 (15.9)	
no	134 (11.3)	21 (17.9)	60 (10.1)	53 (84.1)	
*Definitive closure*					**< 0.001**
Yes	888 (75.1)	102 (82.3)	724 (76.3)	62 (56.4)	
No	295 (24.9)	22 (17.7)	225 (23.7)	48 (43.6)	
*Fascial closure*					**< 0.001**
Yes	773 (65.3)	92 (90.2)	638 (88.1)	43 (69.4)	
No	115 (9.7)	10 (9.8)	86 (11.9)	19 (30.6)	
EAF*					0.134
Yes	29 (2.5)	5 (4.9)	20 (2.8)	4 (6.6)	
No Missing	859 (72.6) 295 (24.9%)	97 (95.1)	704 (97.2)	58 (93.5)	
*Prosthesis*					**< 0.001**
Yes	129 (10.9)	2 (2.0)	125 (17.3)	2 (3.2)	
No	759 (64.2)	100 (98.0)	599 (82.7)	60 (96.8)	
*Kind of prosthesis*					**< 0.001**
Biological	78 (6.6)	1 (1.0)	77 (10.6)	0	
Composite	3 (0.3)	0	3 (0.4)	0	
Not resorbable	14 (1.2)	1 (1.0)	12 (1.7)	1 (1.6)	
Resorbable	34 (2.9)	0	33 (4.6)	1 (1.6)	
No prosthesis	759 (64.2)	100 (98.0)	599 (82.7)	60 (96.8)	

Bold characters indicate statistically significant values

TACT—temporary abdominal closure technique; NPWT—negative pressure wound therapy; EAF—entero-atmospheric fistula

*Missing information of 295 patients

**Fig. 4:**
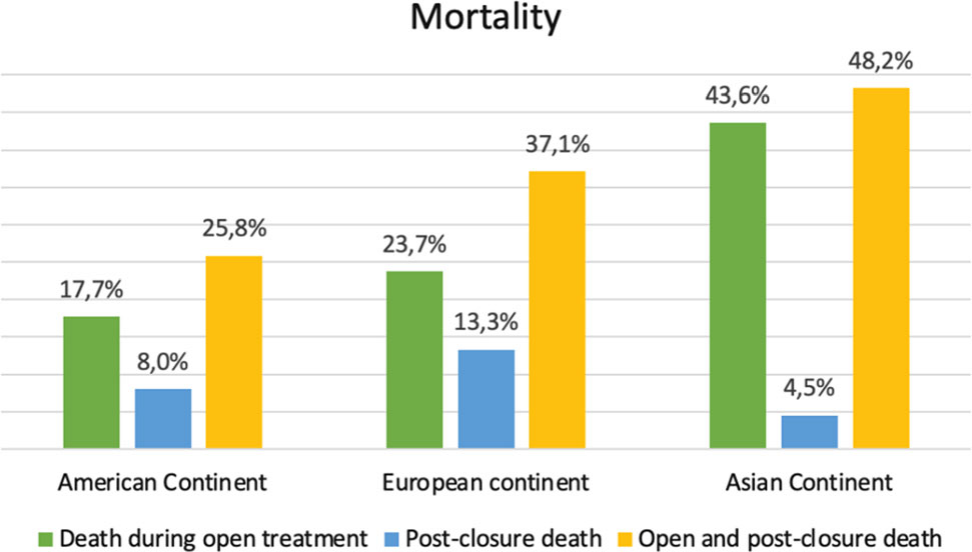
Mortality data through the different continents

As shown in Table 4, the median duration of open abdomen was 4 days (IQR 2–7) with the Asiatic continent deviating from these data with a median of 7 days (IQR 3–18) (*p* < 0.001). Intensive care unit (ICU) length of stay (LOS) and hospital LOS was 8 and 11 days, respectively, without significant differences between continents. Overall morbidity and mortality rates for American, European and Asiatic Continent were, respectively, 75.8%, 75.3%, 91.8% (*p* = 0.001) and 31.9%, 51.6%, 56.9% (*p* < 0.001, Fig. 4). Nevertheless, the complications rate after the definitive closure was not significant different among continents (Table 5).

**Table 4:** Outcomes

	Total N = 1183	American continent N = 124 (10.5)	European continent N = 949 (80.2)	Asiatic continent N = 110 (9.3)	*P* value
Open time	4 (2–7)	4 (2–7)	4 (2–7)	7 (3–18)	**0.001**
ICULOS	8 (4–17)	10 (3–22)	7 (4–16)	9 (5–19)	0.115
HLOS	11 (6–21)	16 (7–30)	10 (6–20)	12 (7–20)	0.033
Death during open treatment	295 (24.9)	22 (17.7)	225 (23.7)	48 (43.6)	**< 0.001**
Post-closure death	142 (12.0)	10 (8.0)	127 (13.3)	5 (4.5)	0.028
Open and post-closure death	437 (36.9)	32 (25.8)	352 (37.1)	53 (48.2)	**0.002**
Overall complications	910 (76.9)	94 (75.8)	715 (75.3)	101 (91.8)	**0.001**
Mortality at 1 month	54 (4.6)	3 (3.8)	48 (9.7)	3 (6.3)	0.205
Mortality at 1 year	44 (3.6)	2 (8.0)	40 (21.2)	2 (28.6)	0.206
Overall mortality	535 (43.8)	37 (31.9)	440 (51.6)	58 (56.9)	**< 0.001**
Incisional hernia	50 (4.2)	5 (6.3)	39 (7.8)	6 (12.5)	0.420

Bold characters indicate statistically significant values

ICULOS—intensive care unit long of stay; HLOS—hospital long of stay

**Table 5:** Complications

	Total N = 1183	American continent N = 124 (10.5)	European continent N = 949 (80.2)	Asiatic continent N = 110 (9.3)	*P* value
*Complications during open treatment*					**< 0.001**
Yes	766 (64.8)	86 (69.4)	580 (61.1)	100 (90.9)	
No	417 (35.2)	38 (30.6)	369 (38.9)	10 (9.1)	
Anastomosis dehiscence	53 (4.5)	8 (6.5)	37 (3.9)	8 (7.3)	0.118
Bleeding	169 (14.3)	20 (16.1)	130 (13.7)	19 (17.3)	0.488
Myocardial infarction	23 (1.9)	5 (4.0)	14 (1.5)	4 (3.6)	0.044
Deep venous thrombosis	7 (0.6)	1 (0.8)	5 (0.5)	1 (0.9)	1.000
Pulmonary embolism	7 (0.6)	2 (1.6)	4 (0.4)	1 (0.9)	0.144
Arithmia and other cardiological complications	139 (11.7)	22 (17.7)	104 (11.0)	13 (11.8)	0.091
Sepsis	150 (12.7)	17 (13.7)	109 (11.5)	24 (21.8)	**0.008**
Pneumonia and ventilator dependence	188 (15.9)	26 (21.0)	119 (12.5)	43 (39.1)	**< 0.001**
*Post-closure complications*					0.169
Yes	555 (46.9)	71 (12.8)	442 (61.0)	42 (67.7)	
No	333 (28.1)	31 (30.4)	282 (39.0)	20 (32.3)	
Bleeding	69 (5.8)	6 (5.9)	60 (8.3)	3 (4.8)	0.590
Myocardial infarction	11 (0.9)	3 (2.9)	8 (1.1)	0	0.229
Deep venous thrombosis	5 (0.4)	1 (1.0)	4 (0.6)	0	0.641
Pulmonary embolism	3 (0.3)	0	3 (0.4)	0	1.000
Arithmia and other cardiological complications	74 (6.3)	9 (8.8)	65 (9.0)	0	0.048
Peritonitis/intra-abdominal abscess	43 (3.6)	0	9 (1.2)	0	0.798
Pneumonia and ventilator dependence	176 (14.9)	17 (16.7)	143 (19.8)	16 (25.8)	0.363
Wound infection	110 (9.3)	18 (17.6)	74 (10.2)	18 (29.0)	**< 0.001**

Bold characters indicate statistically significant values

## Discussion

The present analysis highlights an heterogeneous mix of patients, indications, and treatments across different continents, leading to interesting differences. American patients were younger than in Europe, where patients were older with major comorbidities such as cardiomyopathy and pneumological disorders. Feasibility of OA treatment has been demonstrated at every age and age alone cannot be considered a determinant for patient’s selection. Considering the BMI, American, European, and Asiatic patients were not different. However, it has been demonstrated that application of the current World Health Organization BMI cut-off points underestimates obesity-related risks of Asiatic populations [9]. Recent data showed that at the same BMI, Asians had more than double risk of developing type 2 diabetes, hypertension, and cardiovascular disease than their European counterparts. For this reason, obesity for Asiatic population is considered at a BMI value greater than 25. In this study the median BMI of Asiatic patients was 26 and 68 (61.8%) were obese. The association between obesity and adverse associated to OA as higher morbidity and mortality, longer ICU and hospital stay may partially explain the different overall outcomes found in the different continents [5]. Moreover, the prevalence of ASA score ≥ 3 in Europe and Asia suggests that amore unfavorable characteristics of these patients respect American ones with, consequently, greater probability of develop complications related to OA.

Almost half of OA recorded in IROA from the three continents was performed for peritonitis and abdominal sepsis. The highest percentage of patients treated for peritonitis was in the European continent (53.3%) confirming existing data [10] even if controversies around this indication exist [11]. In the American continent, trauma patients represented the second most common cause of OA and it was performed in a similar percentage of cases of peritonitis [12, 13]. The use of OA in case of peritonitis may help in controlling any persistent source of infections, more effectively remove pro-inflammatory cytokines situated in the peritoneal fluid, provide prophylaxis against development of the abdominal compartment syndrome, and allow for safe deferred gastrointestinal anastomosis [14]. A randomized controlled trial (RCT) the Closed or Open after Laparotomy (COOL) study was launched to assess whether it is better to close the abdomen or to keep it open with NPWT in severe abdominal sepsis patients [15].

As outlined by Balogh et al., cases of ACS requiring decompressive laparotomy are becoming increasingly rare thanks to the avoidance of over-resuscitation or active application of de-resuscitation [16]. Results from IROA confirmed this data since ACS has been an indication for laparotomy in only 3.7% of cases especially in Asiatic continent, 29.8% of patients presented with grade I of IAH. Instead Brandon et al. reported a 6% of laparotomy due to ACS [17]. However, it must be observed that routine assessment of intra-abdominal pressure is not so diffused.

The 2018 WSES guidelines recommended using NPWT with continuous fascial traction as prefer technique and TACT without negative pressure only in low resource settings [8]. However, preliminary data from IROA demonstrated that, in patients affected by peritonitis, NPWT is the most effective in reducing mortality rate and complications. This could be explained by the presence of inflammatory ascites, which has a central role in sepsis [18]. Indeed animal studies suggest that TACT that employ negative pressure to the peritoneal cavity may remove inflammatory ascites, reducing passage of cytokines (TNF, IL6, IL1B, IL12) to the systemic circulation leading to less histologic damage in the lungs, kidneys, liver, intestines, and preventing multiorgan dysfunction [19, 20]. Conversely, in trauma patients, TACT without negative pressure improves survival and definitive closure outcomes as well [2]. Despite of these evidences, in American continent, which has the highest percentage of trauma, the non-negative pressure system is utilized in only 5.6% of cases. Interestingly, in the Asiatic continent, where peritonitis is the first indication, non-commercial NPWT is mostly used. It is important to keep in mind that each technique has a different efficacy in removing intra-abdominal toxin or bacteria-rich fluids and pro-inflammatory cytokines [21]. Moreover, using the most appropriate TACT in different clinical situations may influence the time of abdominal closure [2, 22]. In America, more than 80% of patients reached abdominal closure and almost all with fascial closure. In the Asiatic continent only 56% of patients reached abdominal closure. This can be associated to the different usage of negative pressure techniques. NPWT seems to be associated with improved survival and increased abdominal fascia closure rates when compared with the Barker vacuum pack [23, 24]. Other authors as well showed higher primary fascial closure rates using NPWT in combination with "dynamic closure" technique [25, 26]. Despite these results, IROA study showed that negative pressure associated to dynamic tension was used in 3.6% of cases. No cases were registered in the American continent. The prosthesis has been utilized in 10.9% of cases, and particularly in the European continent (17.3%) where in most cases has been utilized a biological mesh. The use of this type of prosthesis has not been reported in Asia.

IROA study showed that this linear correlation begins earlier from the first OA days increasing progressively from the 5–6 postoperative day [2]. Therefore, early definitive closure should be one of the main aims of the OA management [27]. Asiatic continent had a median OA duration of 7 days (IQR 3–18) with more than 40% of death during treatment and more than 90% of complications. The American continent with equal median of days of treatment of European continent, appeared to be the continent with the lowest rate of death and complication during treatment (Fig. 1). However, it must be observed as the indication to OA differs within different countries. It has been demonstrated that factors associated to the different OA indications may influence the outcomes.

In the Asiatic continent 90% of complications occur during open treatment and 40% of patients suffer from respiratory failure, dependence on the ventilator and sepsis. These complications are present in other continents but with a different rate. It must be considered the different features of patients: in the European and Asiatic continent prevail older and compromised patients with ASA > III. This population is at risk of severe complication due to comorbidities and reduced immunological, nutritional and functional status related to the effects of aging itself [4, 6]. Moreover, many Asiatic patients may be considered obese, and this may contribute to a worse outcome. Europe showed a lower complication rate than Asiatic continent but a major mortality post closure of abdomen.

The incidence of fistula in OA has been reported variously depending on the indication for OA varying from 4.5 to 25 in trauma patients and from 5.7 to 17.2 in non-trauma patients [28]. This latest data from IROA report an incidence of EAF of 2,5% without significant differences among continents. These data confirmed the preliminary results that EAF is not connected with the presence of active suction but, with the duration of the treatment state of nutrition and presence of cancer [7].

## Conclusion

There is no uniformity in OA management in the different continents. Heterogeneous adherence to international guidelines application is evident. Different temporary abdominal closure techniques in relation to indications led to different outcome across the continents. Adherence to guidelines, combined with more consistent data, will ultimately allow improving knowledge and outcomes.

## Data Availability

Not applicable.
